# Probabilistic Model Updating for Sizing of Hole-Edge Crack Using Fiber Bragg Grating Sensors and the High-Order Extended Finite Element Method

**DOI:** 10.3390/s16111956

**Published:** 2016-11-21

**Authors:** Jingjing He, Jinsong Yang, Yongxiang Wang, Haim Waisman, Weifang Zhang

**Affiliations:** 1 School of Reliability and System Engineering, Beihang University, Weimin Building, No. 37, Xueyuan Road, Haidian District, Beijing 100191, China; hejingjing@buaa.edu.cn (J.H.); yangjinsong@buaa.edu.cn (J.Y.); zhangweifang@buaa.edu.cn (W.Z.); 2 Department of Civil Engineering & Engineering Mechanics, Columbia University, New York, NY 10027, USA; waisman@civil.columbia.edu

**Keywords:** FBG sensor, reflection intensity spectra, high-order XFEM, probabilistic crack quantification, Bayesian updating

## Abstract

This paper presents a novel framework for probabilistic crack size quantification using fiber Bragg grating (FBG) sensors. The key idea is to use a high-order extended finite element method (XFEM) together with a transfer (T)-matrix method to analyze the reflection intensity spectra of FBG sensors, for various crack sizes. Compared with the standard FEM, the XFEM offers two superior capabilities: (i) a more accurate representation of fields in the vicinity of the crack tip singularity and (ii) alleviation of the need for costly re-meshing as the crack size changes. Apart from the classical four-term asymptotic enrichment functions in XFEM, we also propose to incorporate higher-order functions, aiming to further improve the accuracy of strain fields upon which the reflection intensity spectra are based. The wavelength of the reflection intensity spectra is extracted as a damage sensitive quantity, and a baseline model with five parameters is established to quantify its correlation with the crack size. In order to test the feasibility of the predictive model, we design FBG sensor-based experiments to detect fatigue crack growth in structures. Furthermore, a Bayesian method is proposed to update the parameters of the baseline model using only a few available experimental data points (wavelength versus crack size) measured by one of the FBG sensors and an optical microscope, respectively. Given the remaining data points of wavelengths, even measured by FBG sensors at different positions, the updated model is shown to give crack size predictions that match well with the experimental observations.

## 1. Introduction

Fatigue cracks emanating from the edge of holes are common dangerous defects in the aircraft industry [1]. Under cyclic loading conditions, the development of fatigue damage due to stress concentration constitutes a safety-critical problem for aging aircraft. To ensure the safety and functionality of such structures, a real-time, low cost, in situ monitoring of fatigue cracks is needed and has been a topic of significant research in structural health monitoring (SHM) in the last few decades [2,3,4,5,6,7,8].

The optical fiber Bragg grating (FBG) sensor has been widely applied to structural health monitoring applications due to several advantages over conventional sensors, such as a small physical size, insensitivity to electromagnetic interference, lightweight, capability of sensing at high temperature and environmentally-unfavorable conditions and multiplexing ability [9]. In order to employ FBG-type sensors for crack detection, one should mount these sensors on the structure in locations that are close to the damaged zones. Due to the complex plastic deformation ahead of the crack tip, there is a significant strain gradient along the grating of FBG sensors, which remarkably affects their reflection intensity spectrum. The underlying mechanism for FBG sensor damage detection is based on changes of the characteristics of the reflection intensity spectrum [10,11,12]. These changes can be used to correlate the damage with crack growth and have been studied experimentally and theoretically. Mizutani [13] proposed a damage index defined as the full width at half maximum (FWHM) of the FBG reflected spectra to measure the extent of transverse cracking in cross-ply laminates. As the laminates were loaded in tension, the FWHM showed a strong correlation to the transverse crack density. Yuan [14] applied a wavelet analysis theory to extract two damage factors, the crack initiation factor (CIF) and the crack propagation factor (CPF), for damage initiation and propagation degree identification. Bernasconi [15] investigated experimentally a fatigue crack growth in adhesively-bonded joints of thick composite laminates by employing an array of FBG sensors. The transfer matrix method (T-matrix) was firstly introduced by Leblanc [16] to simulate the reflection intensity spectrum of FBG according to the strain profile along the grating. Huang [17] developed an optimization approach for crack damage reconstruction based on the simulated reflection intensity spectrum through the T-matrix method.

Despite the number of approaches proposed in the literature for crack detection using FBG sensors, quantitative evaluation of the crack size using direct FBG sensors measurements is not common. One of the major difficulties is that the correlation of the physical model and the reflection intensity spectrum for varying crack sizes changes with structure geometries and working conditions. Hence, a model obtained for a specific target is not generic and cannot be directly employed for other geometry or loading configurations, as it may lead to unreliable results. Therefore, parametric crack detection experiments using FBG sensors must first be done in order to establish the crack size quantification method for each specific target system. However, it may not be practical to conduct such testing for a wide range of applications due to time and economic constraints for real engineering applications.

To address this issue, efforts have been made to calculate the strain distribution along the grating using the finite element method (FEM), rather than experimentally measuring the strain field using sensor techniques, such as strain gage and FBG sensors [18,19,20]. FEM was employed for example by Mckenzie et al. [21] to simulate the crack tip strain profile of an aluminum component bonded with a boron-epoxy unidirectional composite patch. The simulated strain results were used for configuration optimization of an FBG array. For crack tip location identification purposes, Sans [11] calculated the axial strain profile using FEM of the long embedded FBG sensors in carbon/epoxy unidirectional samples.

However, obtaining accurate fracture mechanics solutions by a conventional FEM is computationally expensive since the mesh must conform to the crack geometry, and a high level of refinement is generally required in the vicinity of the crack tip. For simulation-based crack size quantification methods, the inherent limitation of the standard FEM becomes even severer since numerous FE analyses are needed to extract strain profiles along gratings and reflection intensity spectra corresponding to different crack sizes. To alleviate the computational burden of multiple reflection intensity spectrum simulations, the extended finite element method (XFEM), originally proposed by Belytschko and his coworkers [22,23], is combined for the first time with FBG sensors in this paper for crack size quantification. The XFEM enhances the solution space of standard FEM with discontinuous and asymptotic functions via a local partition-of-unity (PU) method [24]. Compared with the standard FEM, the XFEM offers two superior capabilities: (i) a more accurate representation of fields in the vicinity of the crack tip singularity and (ii) alleviation of the need for costly re-meshing as the crack is propagating in the structure [25,26]. These favorable features have been exploited in [27,28,29,30,31] by combining XFEM and optimization algorithms for deterministic and probabilistic flaw detection in structures. In [31], the Bayesian approach [32,33,34,35] was used to quantify uncertainties from modeling errors and measurement noise, leading to the probability distributions of the flaw parameters. Since the extracted reflection intensity spectrum relies heavily on the accuracy of the strain field, we propose to use not only the leading, but also the higher-order terms of the Williams asymptotic solution [36] as the enrichment functions. While the idea of incorporating the high-order enrichment was explored in the previous studies [37,38,39] mainly for direct extraction of stress intensity factors, the motivation here is to effectively improve the accuracy of the simulated strain field and, in turn, the reflection intensity spectrum.

Another difficulty of crack size quantification is the uncertainty in the parameters of real structures, which are typically not captured by deterministic models, such as a standard FEM analysis. For realistic engineering applications, uncertainty in crack characterization may arise due to manufacturing conditions, sensor installation variability, specimen geometry, working conditions, environmental noise, operational errors, etc. Hence, direct correlation of the crack size FEM model and real structures will yield unreliable results. This is a challenging practical problem that requires the capability of model parameter tuning with an appropriate uncertainty quantification.

To resolve the above difficulties in modeling and sizing of the hole-edge crack using FBG sensors, three major efforts are made in this paper: (1) a high-order XFEM is proposed to construct the strain profile ahead of the crack tip. Compared with the conventional XFEM method, the high-order XFEM has been shown to give more accurate displacement/strain/stress fields, especially when the observation point (FBG sensor) is close to the crack tip. (2) An appropriate model is proposed to describe the correlation between the FBG measurements and crack size; and (3) a Bayesian updating method is proposed to incorporate the experimental data for probabilistic crack quantification. In this paper, the high-order XFEM and Bayesian method are combined to form a general procedure for probabilistic hole-edge crack size quantification.

This paper is organized as follows. First, the framework of the proposed probabilistic crack size quantification method is introduced. Next, the FBG reflection intensity spectra simulation method is introduced on the basis of the XFEM and the T-matrix method. Following this, the simulation results are used to establish the baseline crack size quantification model and to construct the prior distribution of model parameters. The experimental measurement data from the fatigue testing on an aluminum alloy component are employed to update the baseline model parameters using the Bayesian method and to obtain their posterior estimates. The influence of measurement location is also investigated, and conclusions summarizing this research are drawn.

## 2. Methodology Development

**Figure 1:**
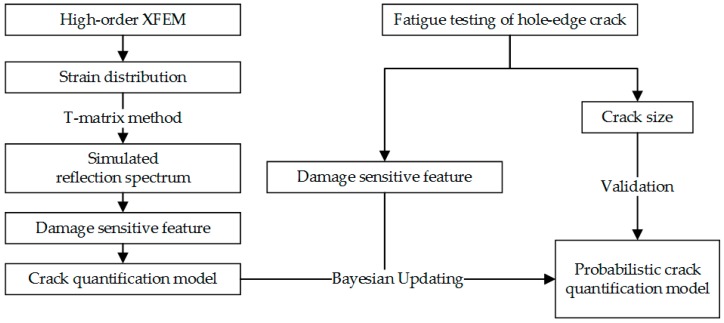
The flow chart of hole-edge crack quantitative detection.

The objective of this study is to develop an overall methodology for reliable, efficient and accurate hole-edge crack quantification using FBG sensors. Figure 1 presents the flowchart used to carry out this work. Initially, the XFEM is applied to model the strain profiles for various hole-edge crack sizes. Subsequently, the corresponding reflection intensity spectra are constructed using the T-matrix method based on the strain profiles. A baseline crack size quantification model is developed to correlate the crack size and damage sensitive features extracted from the reflection intensity spectrum. Prior distributions of model parameters are obtained based on the simulation data. For the purposes of parameter updating and model validation, fatigue tests are conducted on specimens with a hole and a pre-crack. FBG sensors are installed on the specimens and are used to collect reflection intensity spectrum data for the crack regions. The resulting experiment data are employed to update the baseline model parameters for more accurate crack size predictions. Following this, a probabilistic crack quantification model for the hole-edge crack can be achieved. In this study, FBG sensors at different locations are also used to investigate the variability effect of crack quantification.

### 2.1. High-Order Extended Finite Element Method

**Figure 2:**
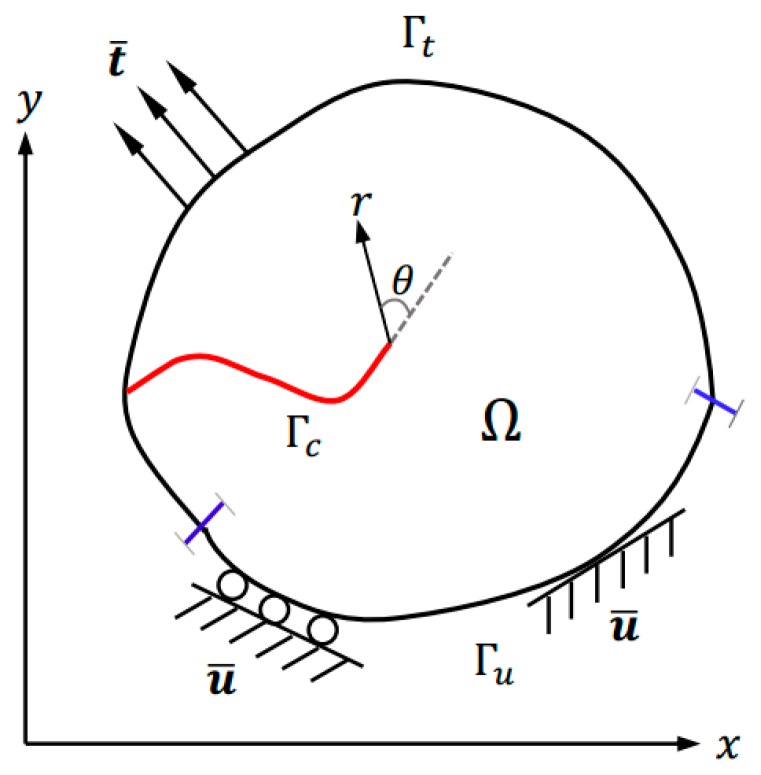
A solid containing a crack represented by the red solid line.

We consider a solid Ω with an external boundary Γ, as shown in Figure 2. The solid contains a traction-free crack, indicated by an internal boundary $\Gamma_{c}$. Prescribed displacements $\bar{u}$ are applied on Dirichlet boundaries $\Gamma_{u}$, whereas surface tractions $\bar{t}$ are imposed on the complementary Neumann boundaries $\Gamma_{c}$, with $\Gamma_{u}\cap \Gamma_{t}=\emptyset$ and $\bar{\Gamma_{u}\cup \Gamma_{t}}=\Gamma$.

The Galerkin approximation of the proposed problem is to seek a kinematically-admissible displacement field $u^{h}\in U$, which is a finite-dimensional subspace of the solution space, such that:

(1) $$\int_{\Omega }\varepsilon (u^{h}):C:\varepsilon (w^{h})d\Omega =\int_{\Gamma_{t}}\bar{t}\cdot w^{h}d\Gamma ,\;\forall w^{h}\in U_{0}$$

where ε and C are the standard strain and elasticity tensor, respectively. The weighing functions $w^{h}$, whose values vanish on $\Gamma_{u}$, belong to the finite-dimensional subspace $U_{0}$.

In the XFEM, the standard FE polynomial approximation space is augmented with a set of enrichment functions according to the physics of the problem at hand. For linear elastic fracture problems, the XFEM displacement approximation $u^{h}$ takes the form:

(2) $$u^{h}(x)=\sum_{I\in S}N_{I}(x)u_{I}+\sum_{I\in S_{H}}N_{I}(x)H(x)a_{I}+\sum_{I\in S_{T}}N_{I}(x)\sum_{a=1}^{n_{f}}f_{\alpha }(x)b_{\alpha I}$$

where x is the spatial coordinate and the standard FE shape functions associated with standard degrees of freedom (DOFs) $u_{I}$ are denoted by $N_{I}(x)$. The set of all nodes in the domain is represented by S. The basis function support of the node set $S_{H}$ is entirely split by the crack, whilst $S_{T}$ contains nodes with the crack tip located in the support of their basis functions. The nodal DOFs $a_{I}$ and $b_{\alpha I}$ correspond to the enrichment functions H and $f_{\alpha }$, respectively. The number of adopted near-tip asymptotic enrichment functions is denoted by $n_{f}$. The generalized Heaviside function H(x) captures the displacement jump across the crack surface:

(3) $$H(x)=\left\{ \begin{array}{ll} +1 & \mathrm{above}\;\Gamma_{c}\\ -1 & \mathrm{below}\;\Gamma_{c} \end{array}\right.$$

The crack-tip branch functions $f_{\alpha }(x)$ are determined from the asymptotic solution of Williams [36] for the near-tip displacement field. These functions are defined in terms of the polar coordinates (r,θ) with the origin at the crack tip, as illustrated in Figure 2. While most researchers only use the leading terms in the asymptotic solution indicated by Equation (4), herein, we also incorporate higher-order terms Equations (5)–(7) (up to $r^{2}$). The following full set of branch functions $F(r,\theta )=\{F^{1},F^{2},F^{3},F^{4}\}$ with 13 terms is used in our study:

(4) $$\sqrt{r}:\;F^{1}=\{f_{1},f_{2},f_{3},f_{4}\}=\sqrt{r}\{\sin\frac{\theta }{2},\cos\frac{\theta }{2},\sin\theta \sin\frac{\theta }{2},\sin\theta \cos\frac{\theta }{2}\}$$

(5) $$r:\;F^{2}=\{f_{5},f_{6}\}=r\{\sin\theta ,\cos\theta \}$$

(6) $$r^{1.5}:\;F^{3}=\{f_{7},f_{8},f_{9},f_{10}\}=r^{1.5}\{\sin\frac{\theta }{2},\cos\frac{\theta }{2},\sin\theta \sin\frac{\theta }{2},\sin\theta \cos\frac{\theta }{2}\}$$

(7) $$r^{2}:\;F^{4}=\{f_{11},f_{12},f_{13}\}=r^{2}\{1,\sin2\theta ,\cos2\theta \}$$

For a detailed derivation of these enrichment functions, the interested reader is referred to our previous work [38].

**Figure 3:**
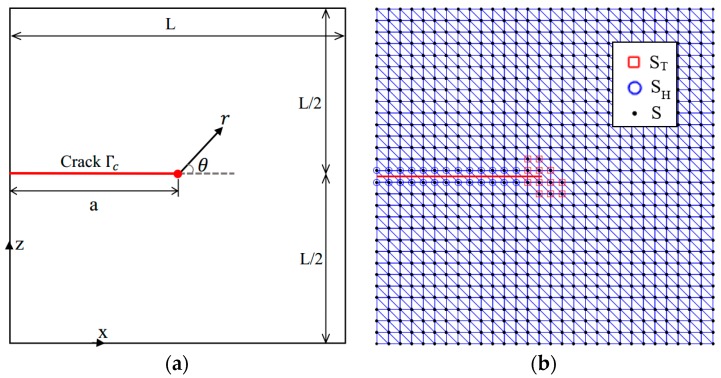
A square plate with an edge crack: (**a**) geometry; (**b**) a finite element mesh with 30 × 30 nodes. Tip-enriched nodes $S_{T}$ are indicated by red squares, where blue circles denote Heaviside-enriched nodes $S_{H}$.

An illustrative example is presented in the following to demonstrate that the higher-order enrichment terms can serve as an alternative to strain/stress recovery techniques [40,41,42] to obtain highly accurate fields, especially when the observation point (FBG sensor) is close to the crack tip. Let us consider the square plate Ω of edge length *L* = 10 mm with an edge crack of a=5 mm, as shown in Figure 3. The Williams asymptotic displacement solution, with a Mode I stress intensity factor of one, is imposed on the boundary Γ. We have considered a state of plane strain, with Young’s modulus $E=10^{7}$ MPa and Poisson’s ratio υ=0.3. The domain is discretized using a uniform triangular mesh with 30 × 30 nodes, as shown in Figure 3b. The enriched nodes are also marked in this figure.

**Figure 4:**
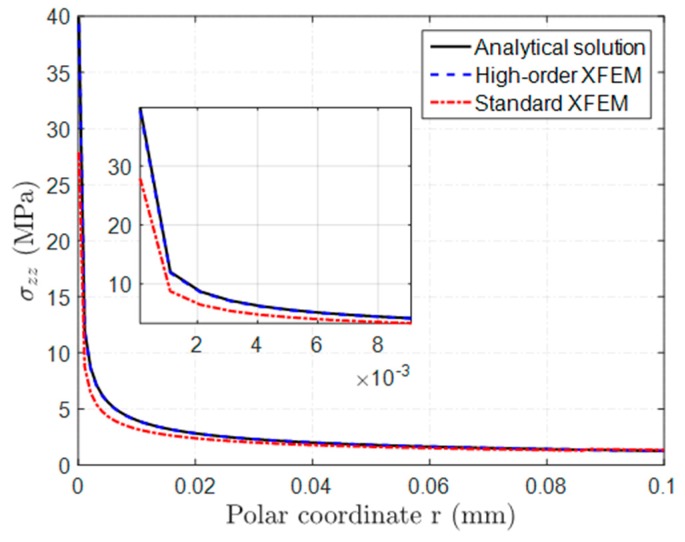
Comparison of stress profiles ahead of the crack tip between regular and high-order XFEM. High-order XFEM leads to very accurate results.

The normal stress $\sigma_{yy}$ distribution ahead of the crack tip is depicted in Figure 4 for the regular and high-order XFEM. The exact solution is also plotted for reference. As can be seen, the high-order stress profile almost coincides with the exact solution, whereas a significant discrepancy between the regular XFEM solution and the exact one is observed. In addition, the relative error of XFEM solutions is reported in Table 1 for some representative points. It can clearly be seen that the relative error of high-order result is less than 3%, which is much lower than the error (up to 27%) of regular XFEM in this case.

### 2.2. T-Matrix Method

The transfer matrix method (T-matrix method) is used to construct the reflection intensity spectrum of FBG from the strain profile along the grating obtained through XFEM. The T-matrix formulation approximates the applied strain as a piecewise constant function and calculates the average period in each grating segment due to the applied strain. Substituting this local period back into the coupled equations leads to the output fields along the entire grating [43,44]. Assuming that a grating of length *L is* subdivided into *M* segments, each segment with an effective, constant period, $\Lambda_{i}$, is given as:

(8) $$\Lambda_{i}=\Lambda_{0}\left(1+a\varepsilon_{zz}\right)$$

where $a=1-\frac{1}{2}n_{eff0}^{2}\left[p_{12}-\nu (p_{11}-p_{12})\right]$ is the grating gauge factor [45], in which $p_{11}$ and $p_{12}$ are the components of the fiber-optic strain tensor and ν is the Poisson ratio. The axial coordinate of the fiber is represented as z, and the limits of the grating are defined between −L/2≤z≤L/2. The initial grating period is denoted by $\Lambda_{0}$, and the initial average effective mode index of refraction at the strain-free state is denoted by $n_{eff0}$. The normal strain averaged over the length of the *i*-th segment is represented as $\varepsilon_{zz}$. Based on mode-coupling theory, the optical transfer matrix for each grating segment results in a 2×2 *T*-matrix that relates the input, transmission and reflection field amplitudes, $T_{i}$,

(9) $$\left[\begin{array}{c} R_{i}\\ S_{i} \end{array}\right]=T_{i}\cdot \left[\begin{array}{c} R_{i-1}\\ S_{i-1} \end{array}\right]$$

where $R_{i}$ and $S_{i}$ are the field amplitudes of the forward and backward guided modes propagating through the *i*-th segment, respectively. The elements of $T_{i}$ have the following full expression [46]:

(10) $$T_{i}=\left[\begin{array}{cc} \cosh\left(\gamma \Delta z\right)-i\frac{\hat{\sigma }}{\gamma }\sinh\left(\gamma \Delta z\right) & -i\frac{\kappa }{\gamma }\sinh\left(\gamma \Delta z\right)\\ i\frac{\kappa }{\gamma }\sinh\left(\gamma \Delta z\right) & \cosh\left(\gamma \Delta z\right)+i\frac{\hat{\sigma }}{\gamma }\sinh\left(\gamma \Delta z\right) \end{array}\right]$$

where Δz is the length of the grating segment. The general ‘dc’ self-coupling coefficient $\hat{\sigma }$ and the ‘ac’ coupling coefficient κ in the *i*-th section are defined as:

(11) $$\hat{\sigma }=\frac{2\pi }{\lambda }\left(n_{eff0}+\bar{\delta n_{eff}}\right)-\frac{\pi }{\Lambda_{i}}$$

(12) $$\kappa =\frac{\pi }{\lambda }\upsilon \bar{\delta n_{eff}}$$

where $\bar{\delta n_{eff}}$ is the ‘dc’ index change spatially averaged over a grating period. The function γ is defined as:

(13) $$\gamma =\sqrt{\kappa^{2}-{\hat{\sigma }}^{2}}$$

The coupling coefficients κ and $\hat{\sigma }$ are locally fixed values of the *i*-th segment. Thus, with all of the T-matrices for the individual segment, the overall amplitudes are calculated as:

(14) $$\left[\begin{array}{c} R_{-L/2}\\ S_{-L/2} \end{array}\right]=T\cdot \left[\begin{array}{c} R_{L/2}\\ S_{L/2} \end{array}\right]$$

with the T-matrix of the entire grating:

(15) $$T=T_{M}T_{M-1}\cdots T_{1}$$

To calculate the reflectivity, the physical boundary conditions $S_{L/2}=0$ and $R_{-L/2}=1$ are imposed to solve Equation (15) [47]. The reflectivity ρ can be calculated as:

(16) $$\rho ={\left|\frac{S_{-L/2}}{R_{-L/2}}\right|}^{2}={\left(\frac{T_{21}}{T_{11}}\right)}^{2}$$

It is well known that the FBG refection intensity spectrum obtained from the T-matrix method is accompanied by a series of side-lobes adjacent to the Bragg wavelength. FBG with the apodization profile of the refractive index is reported to efficiently decrease undesirable side-lobes [48]. The raised cosine apodization function g(z) used in this paper is expressed as:

(17) $$g\left(z\right)=\frac{1}{2}\left[1+\cos\left(\frac{z\pi }{L}\right)\right]$$

The T-matrix method can be adjusted to accommodate the apodization of an FBG sensor by replacing the $\bar{\delta n_{eff}}$ with the following function:

(18) $$\bar{\delta^{\prime }n_{eff}}=\bar{\delta n_{eff}}\cdot g\left(z\right)$$

## 3. Fatigue Crack Size Quantification Using XFEM and the T-Matrix Method

### 3.1. Structural-Optical Simulation

**Figure 5:**
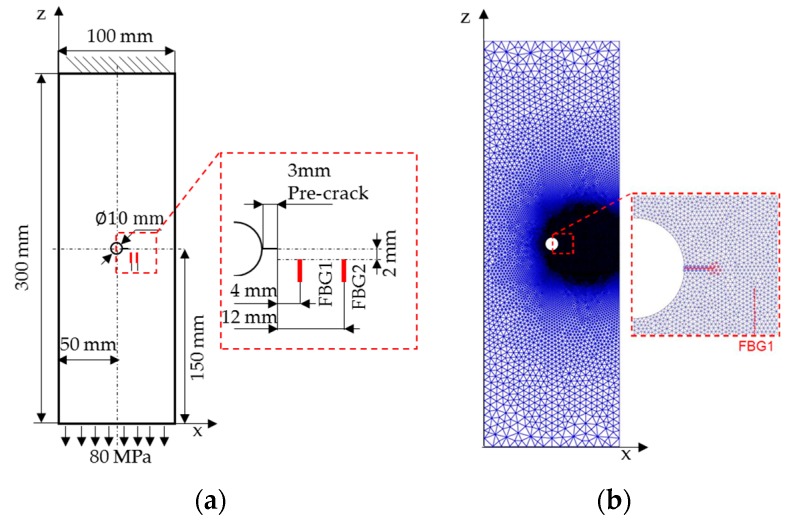
A perforated plate with a crack emanating from the hole-edge: (**a**) geometry, boundary conditions and the layout of FBG sensors; (**b**) FE mesh with FBG Sensor #1 shown as the red dashed line.

The target system studied in this paper is a plate made of aluminum alloy 7075-T6, with the dimensions of 300 mm × 100 mm × 2 mm, as shown in Figure 5a. The properties of the specimen are listed in Table 2. A hole of 10 mm in diameter is in the center of the plate. A pre-crack with a size of 3 mm is introduced by electric discharge machining to trigger fatigue crack propagation. The top boundary is fixed, and a uniform tensile loading of 80 MPa is applied on the bottom. As shown in Figure 5a. Two FBG sensors are glued uniformly onto the specimen surface using liquid cyanoacrylate adhesive. The lengths of FBG1 and FBG2 sensors are 10.2 mm and 10.6 mm, respectively. The horizontal distances from FBG1 and FBG2 to the hole-edge are 7 mm and 15 mm, respectively. In order to sense the change of the axial strain profile due to the crack growth, the axial distance between the FBG sensors and the crack is set to be 2 mm. It bears emphasis that only the reflection intensity spectra of FBG1 are simulated by the XFEM and T-matrix method. The simulated reflection intensity spectra are then used to establish the baseline crack size quantification model and to construct the prior distribution of model parameters. On the other hand, the FBG2 measurements are used for validation purposes, and no structural-optical simulation is performed for this sensor.

**Figure 6:**
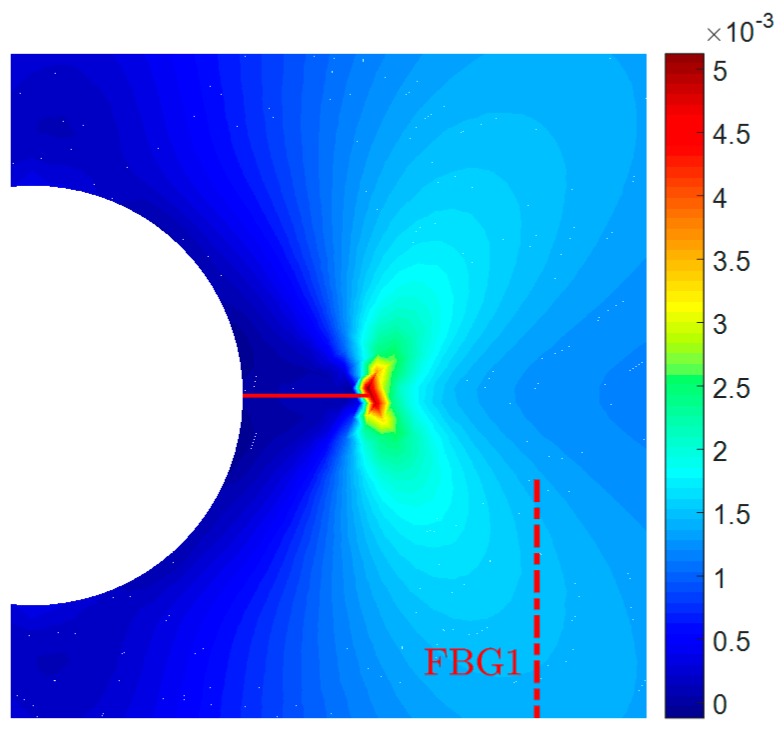
Contour plot of $\varepsilon_{zz}$ around the crack. The corresponding crack size is 3 mm.

The domain is discretized using a mesh consisting of 49,225 linear triangular elements, as shown in Figure 5b. The mesh is refined in the right central part of the specimen where the crack growth is expected. The high-order XFEM is employed to compute the strain distribution along the FBG1 sensor for various crack sizes, ranging from 3–36 mm. In Figure 6, we depict the distribution of strain $\varepsilon_{zz}$ around the crack, for the initial crack size of 3 mm.

**Figure 7:**
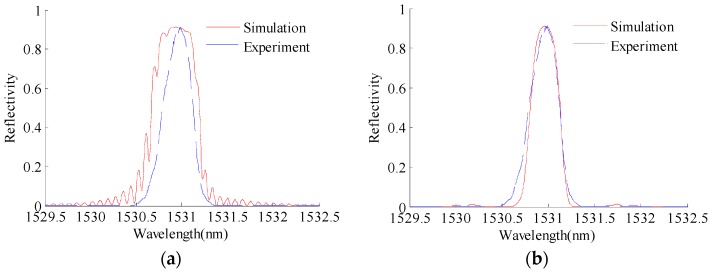
Comparison between the simulated and experimental reflection intensity spectra of FBG1 for the structure with the initial 3-mm pre-crack: (**a**) without the raised cosine apodization function; (**b**) with the raised cosine apodization function.

The T-matrix method is used to construct the reflection intensity spectrum of FBG from the strain profile along the grating obtained through XFEM. Figure 7 represents a comparison between the simulated reflection intensity spectrum of FBG1 without the apodization function and the simulated reflection intensity spectrum with the apodization function for the specimen with a 3-mm pre-crack. The filtering apodization function g(z) vanishes smoothly at the edges of grating. It can be seen that nearly all of the side lobes have been eliminated. In this paper, the experimental reflection intensity spectrum of apodized FBG sensors is collected by an optical sensing interrogator (sm125, Micro Optics Inc., Hackettstown, NJ, USA). As shown in Figure 7b, the simulated reflection intensity spectrum agrees well with the experimental reflection intensity spectrum.

### 3.2. Extraction of a Damage Sensitive Quantity

**Figure 8:**
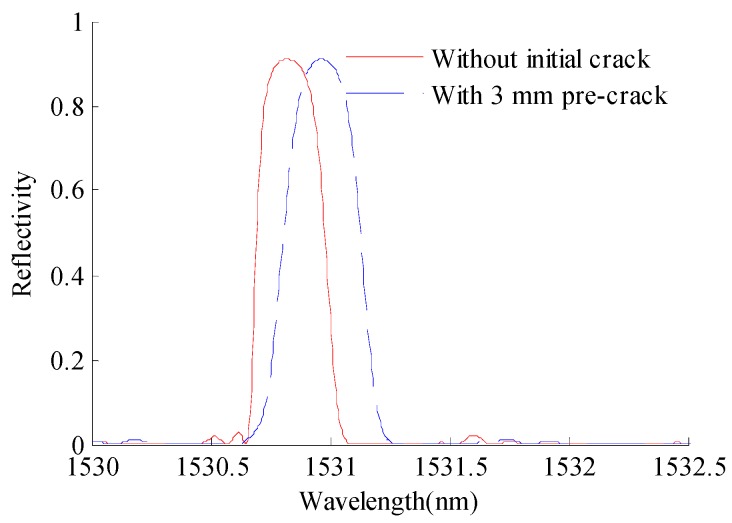
Comparison between the simulated reflection intensity spectra of FBG1 without the initial crack and with the 3-mm pre-crack.

Direct usage of the measured reflection intensity spectrum of FBG sensors for estimating crack size is difficult, and data interpretation is generally required when extracting features that are sensitive to crack sizes. The cross-correlation coefficient, which is a quantitative measure of ‘overlap’ between the measured FBG reflection intensity spectrum and a reference spectrum for the same FBG was proposed by Park [49]. The correlation coefficient is sensitive to both the effects of spectral broadening and increased spectral complexity. However, it was also shown in Park’s study that the cross-correlation coefficient might be contaminated by measurement noise. Okabe [50] found the spectrum width at the half maximum reflectivity to be a good indicator for the quantitative evaluation of the transverse crack density in composite laminates. The damage sensitivity of the spectral bandwidth decreases rapidly as the defect moves away from the FBG sensor [49]. Thus, the spectral bandwidth is not suitable for crack propagation monitoring. It has been reported that the wavelength of the reflection intensity spectrum is sensitive to the magnitude change of the strain component along the axis of the optical fiber due to crack growth [51]. As shown in Figure 8, an obvious wavelength shift can be observed between the simulated reflection intensity spectrum of FBG1 for the specimen without the initial crack and with the 3-mm initial crack. Therefore, the wavelength is selected as a damage-sensitive feature for crack quantification in the current investigation.

**Figure 9:**
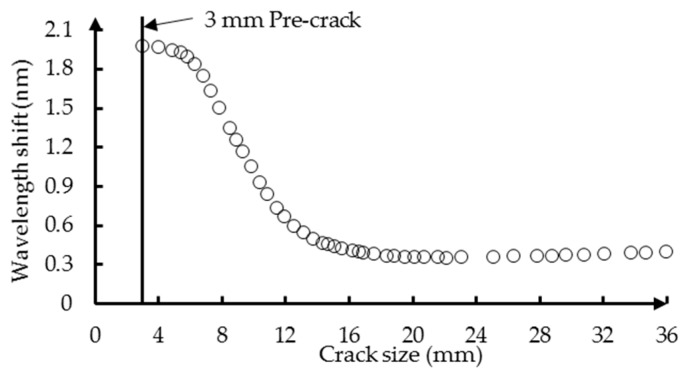
The simulated result: the wavelength shift of FBG1 versus the crack size.

Typically, the maximum detection algorithm is used to accomplish this task by searching for the wavelength corresponding to the maximum power in the reflection intensity spectrum [52]. This algorithm is a pure peak detection approach in the sense that it does not take into account the shape of the spectrum. To this end, Lamberti [44] presents a quadratic interpolation method to compute the wavelength of maximum reflectivity using the following equation:

(19) λmax=argmaxλ{Rp(λ)}

where λ is the wavelength and Rp(λ) indicates the spectrum obtained with a p point quadratic interpolation around the peak wavelength of the original reflection intensity spectrum R(λ). The value of p cannot be too high or too low since that would eliminate some of the benefit introduced by the sub-interpolation function. In this paper, the choice of p = 7 is employed, similar to the recommendation in [44]. Before the fatigue testing, the wavelength $\lambda_{\max}^{ref}$ of the FBG sensor in a free loading state is measured and taken as a reference point. The damage parameter is then measured as the wavelength shift from the reference point and is obtained by subtracting the reference $\lambda_{\max}^{ref}$ from the $\lambda_{\max}$ measured at each crack increment. The crack size versus the wavelength shift of FBG1 is shown in Figure 9 for the problem shown in Figure 5. It can be seen that initially, the wavelength shift decreases significantly with the increase in crack length, but then stabilizes, and a slight increase towards the end of the fatigue life is observed. The metal fracture precipitated by a crack is nearly always preceded by at least a small amount of plastic deformation at the crack tip [53]. The stress concentration in the plastic zone causes a significant strain gradient along the grating of the FBG sensor. The value of strain along the grating reaches the maximum before the crack reaches the FBG sensor location and decreases as the crack moves away from the FBG sensor. The measurement accuracy of the optical sensing interrogator (sm125, Micro Optics Inc., Hackettstown, NJ, USA) is one uε. The corresponding value of wavelength shift is 0.005 nm. In this paper, the specimen was under a static load of 80 MPa when the reflection intensity spectra of the FBG sensors were recorded. The minimum value of the average strain along the FBG sensor is 230 uε. As the crack propagates away from the sensor, the change of wavelength shift becomes smaller.

### 3.3. Quantification Model for Crack Size

**Figure 10:**
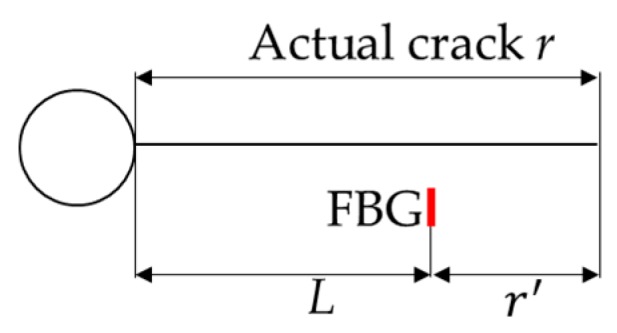
The illustration for the relative crack size $r^{\prime }$

A detection model using the response surface method is introduced in order to quantify the relationship between the crack size and its corresponding wavelength shift. The model is given in Equation (20):

(20) $$\begin{array}{l} \lambda =\frac{p_{1}+p_{2}r^{\prime }+p_{3}{r^{\prime }}^{2}}{1+p_{4}r^{\prime }+p_{5}{r^{\prime }}^{2}}\\ r^{\prime }=r-L \end{array}$$

where λ is the wavelength shift and $p_{1},p_{2}\cdots p_{5}$ are the fitting parameters. The reflection intensity spectrum is affected not only by the size and orientation of the crack itself, but also by its distance to the FBG sensor. In order to incorporate the information of FBG sensor location into this model, $r^{\prime }$ is the relative crack size defined as the physical crack size *r* minus the distance *L* between each FBG sensor and the hole-edge, as shown in Figure 10. Therefore, this model can be easily generalized to different locations of FBG sensors. In this paper, the experiment reflection intensity spectra of FBG2 are used to investigate the influence of sensor location on the detection model. It is worth mentioning that the purpose of the proposed method is to develop a method that can serve as a link between the numerical high-order XFEM method and T-matrix transformation to realistic engineering applications. Therefore, the procedure described in this study is not limited to a particular model (e.g., Equation (20)), and other data-driven models that incorporate more complex physics can also be used.

.

The simulation data from XFEM and the T-matrix method are used to estimate the model parameters, where the coefficients $p_{1},p_{2},p_{3},p_{4},p_{5}$ are obtained through a least square minimization and expressed as:

(21) $$\lambda =\frac{1.698-0.184r^{\prime }+0.016{r^{\prime }}^{2}}{1+0.013r^{\prime }+0.026{r^{\prime }}^{2}}$$

Given a value of wavelength shift, solving the parameterized model gives the corresponding crack size. It should be noted that this quantification model is an idealized model that is based on the numerical model. Nonetheless, realistic engineering applications introduce discrepancies due to uncertainties in crack characterization, manufacturing conditions, sensor installation variability, specimen geometry, working conditions, environmental noise, operational errors, etc., which are not captured in this idealized model. The effectiveness and accuracy of this model for real structures will be verified by the following experimental data.

## 4. Methodology Validation

### 4.1. Experimental Setup

**Figure 11:**
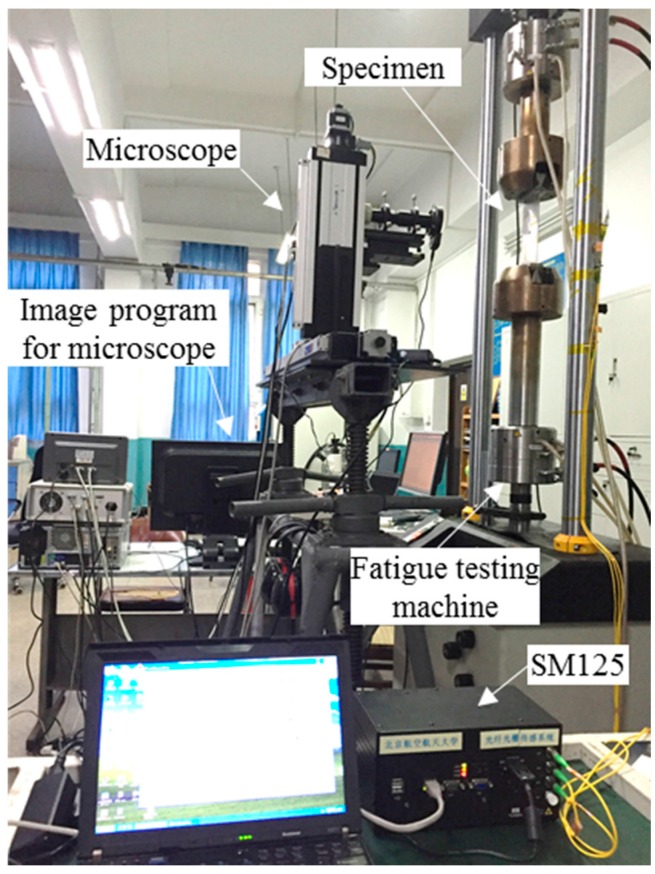
The experimental setup for the hole-edge crack detection.

**Figure 12:**
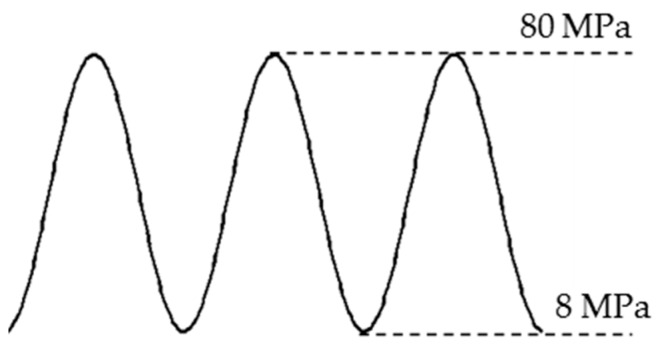
Fatigue loading spectrum for the hole-edge crack specimen.

**Figure 13:**
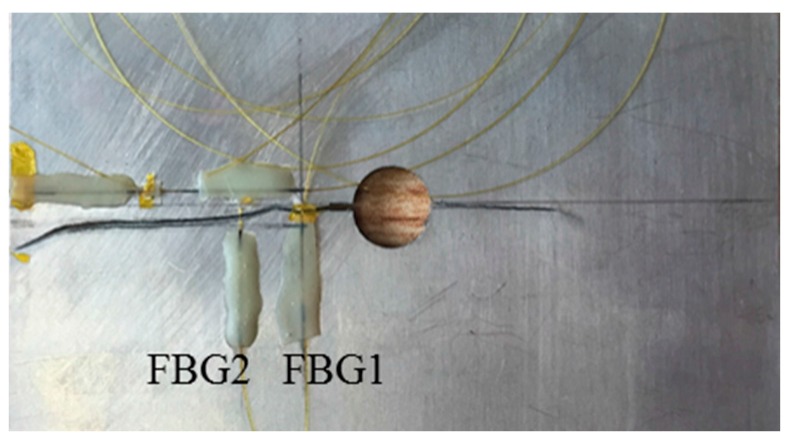
The crack propagation path observed in the experiment. Note the curved path of the crack which introduces additional error from the numerical model.

To validate our methodology, a fatigue crack damage detection experiment platform is developed, and FBG sensors are employed to extract the damage index. The overall experimental setup of the hole-edge crack detection consists of three major parts: the optical sensing system, a fatigue crack measurement system and a fatigue load-cycling system, shown in Figure 11. The reflection intensity spectra of the FBG sensors are measured by an optical sensing interrogator (sm125, Micro Optics Inc., Hackettstown, NJ, USA) with high wavelength accuracy. A traveling optical microscope monitors the fatigue crack size as it grows, with a CCD camera during the loading process. Fatigue testing is conducted using a hydraulic machine (8801, Instron Corporation, Binghamton, NY, USA) with constant fatigue loading along the z-direction in Figure 5a. Figure 12 presents the constant amplitude loading spectra used in this study, with a maximum value of loading set as 80 MPa and a cycling frequency of 5 Hz. In our testing system, the specimen is fully fractured when the crack size reaches 35.9 mm. As shown in Figure 13, the actual crack propagation orientation is not a straight line along the pre-crack, which introduces additional uncertainty in the model.

In the simulation, the crack is assumed to propagate along a straight path, whereas the crack propagation in the experiment is curved. Hence, in real applications, there is additional uncertainty related with crack propagation, which justifies the Bayesian updating procedure proposed in the current work. Before the fatigue testing, the reflection intensity spectra of the FBG sensors under free loading state are measured as a reference. These spectra are used for the inversion of the property parameters of FBG sensors based on the T-matrix method [17]. Properties of FBG sensors are listed in Table 3.

### 4.2. Experimental Results and Damage Sensitivity Index Extraction

**Figure 14:**
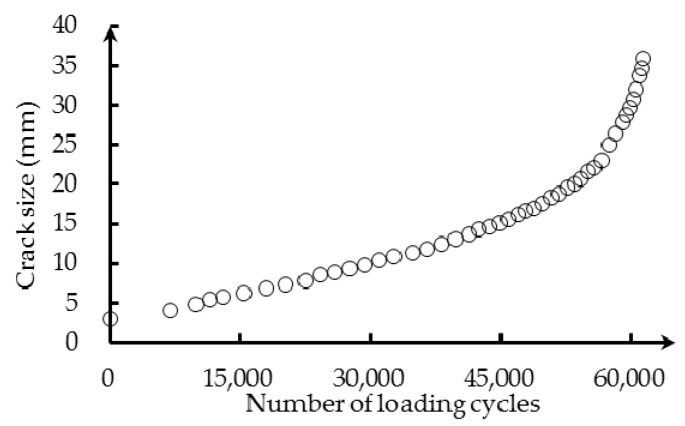
Fatigue testing data.

Figure 14 presents the fatigue testing data. At each of the measurement points, the fatigue loading was paused for data acquisition and crack size measurement. The hydraulic MTS machine imposed a static load of 80 MPa in the structure, and the reflection intensity spectra of the FBG sensors were recorded by sm125. The crack size was measured using microscopic imaging, and the cyclic loading was resumed after data acquisition.

**Figure 15:**
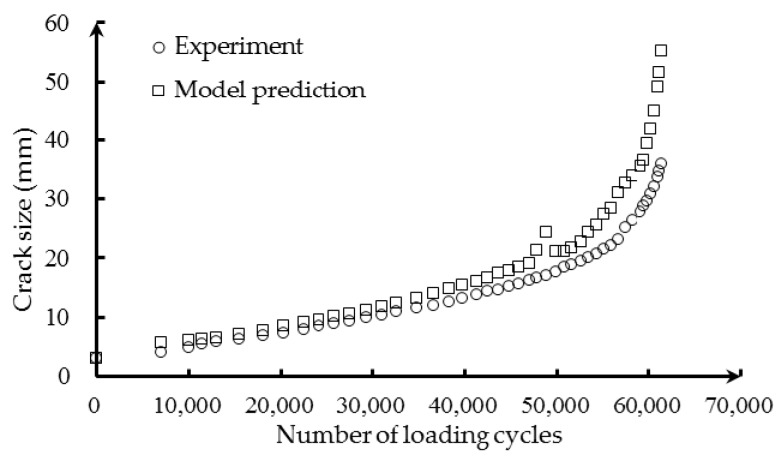
Comparison between the XFEM-based crack size prediction and the actual crack size measurements in fatigue loading.

As mentioned before, the wavelength shift extracted from the measured reflection intensity spectra is employed as a damage sensitive index. The baseline center wavelength is measured when no loading is applied, hence in a loading-free state. The numerical correlation model between the wavelength shift and crack size of Equation (21) is used to predict the crack size based on the measured FBG data. Figure 15 presents the XFEM-based crack size prediction results and the actual crack size measurements. It can be observed that the XFEM-based model prediction yields a relatively larger crack size compared with the experiment. The difference is due to the uncertainty in sensor noise, crack tip orientation, geometry features, as well as quantification modeling error, which is not captured in the numerical deterministic model. To improve the crack size prediction, it is necessary to update the model parameters and reduce the uncertainty. The Bayesian method, employed in our research, is a rational approach for probabilistic model updating and is described as follows.

## 5. Probabilistic Fatigue Crack Sizing Using Experimental Data

The baseline model obtained using the XFEM simulation deviates from the experiments, and therefore, a model updating is necessary. In this section, the Bayesian updating is used for this purpose with added information provided by the FBG1 sensor measurements. The calibrated and updated model is then applied for crack prediction with data obtained by the FBG2 sensor to investigate the influence of sensor location.

### 5.1. Background of the Bayes’ Theorem and Uncertainty Updating

In the aforementioned damage detection problem, the newly-observed information can be incorporated to obtain a better estimation of the system, including its parameter distribution, model accuracy and future performance. Bayes’ theorem has been extensively used in various structural health monitoring applications [54,55,56,57,58]. Bayes’ theorem combines the information of the prior guess with the belief on the current system response through a so-called likelihood function to update the distribution of the parameters of interest [59]. In the Bayesian updating framework, model parameters θ are considered as random variables, which can be updated using in situ observation data $x^{\prime }$. To validate the detection results, the optically-measured crack length is considered as the ground truth. The posterior distribution of the parameters can be express in direct proportion to:

(22) $$q\left(\theta \right)\propto p\left(\theta \right)p\left(x^{\prime }|\theta \right)$$

where p(θ) is the prior distribution of the model parameters θ, where θ can be a vector for multiple parameters or a scalar. $p\left(x^{\prime }|\theta \right)$ is the likelihood function, which reflects the observed current system response $x^{\prime }$ given parameters θ, and q(θ) is the posterior distribution of updated parameters.

For the crack detection problem in this paper, the sources of uncertainties usually include, but are not limited to, model parameters, the measurement data and the damage characterization. Accounting for these uncertainties is essential for detection accuracy and the reliability of the target system. The FE result is more sensitive to structural damage by eliminating the interference of other factors. With the computational efficiency and accuracy of the XFEM in crack propagation modeling, the simulation result is easily obtained. In our approach, the baseline detection model describes the strain profile change due to crack propagation with the corresponding specific physical meanings; therefore, the baseline model can be adapted to realistic engineering applications. However, the uncertainties in measurement data and crack orientation propagate to the detection results. The baseline detection model is not sufficient to directly detect and quantify crack damage for a realistic structure. Hence, the detection model in Equation (20) can be rewritten as:

(23) $$\lambda =M\left(r^{\prime },\theta \right)$$

where θ denotes uncertain model parameter vector $\left(p_{1},p_{2},p_{3},p_{4},p_{5}\right)$that is obtained via statistical regression analysis. Here, λ is the wavelength shift, and $r^{\prime }$ is the relative crack size. In practice, it is necessary to introduce an error term to account for the uncertainties. In our formulation, the relationship between in situ test data $\lambda^{\prime }$ and $M\left(r^{\prime },\theta \right)$ obtained via XFEM and T-matrix simulation is expressed as:

(24) $$\lambda^{\prime }=M\left(r^{\prime },\theta \right)+e$$

Assume that the error term e is a zero mean normal variable [60], expressed as $e\sim N\left(0,\sigma_{e}\right)$. To update the detection model using the measured system response, the likelihood function needs to be constructed. Given *n* groups of measurements, the likelihood function $p\left(x^{\prime }|\theta \right)$ can therefore be expressed as:

(25) $$p\left({\lambda^{\prime }}_{1},{\lambda^{\prime }}_{2},\cdots ,{\lambda^{\prime }}_{n}|\left(\theta ,\sigma_{e}\right)\right)={\left(\frac{1}{\sqrt{2\pi }\sigma_{e}}\right)}^{n}\exp\left\{-\frac{1}{2}\sum_{i=1}^{n}{\left[\frac{\left({\lambda^{\prime }}_{i}-M\left({r^{\prime }}_{i},\theta \right)\right)}{\sigma_{e}}\right]}^{2}\right\}$$

Substituting Equation (25) into Equation (22), the posterior distribution of θ is expressed as:

(26) $$q\left(\left(\theta ,\sigma_{e}\right)|{\lambda^{\prime }}_{1},{\lambda^{\prime }}_{2},\cdots ,{\lambda^{\prime }}_{n}\right)\propto p\left(\theta \right){\left(\frac{1}{\sqrt{2\pi }\sigma_{e}}\right)}^{n}\times \exp\left\{-\frac{1}{2}\sum_{i=1}^{n}{\left[\frac{\left({\lambda^{\prime }}_{i}-M\left({r^{\prime }}_{i},\theta \right)\right)}{\sigma_{e}}\right]}^{2}\right\}$$

The posterior distribution of each parameter is estimated by the samples generated using the Markov chain Monte Carlo (MCMC) method. The posterior belief on crack size is then estimated from the posterior distribution of the updated parameters. A detailed discussion on MCMC is well documented in the open literature [61,62] and is not discussed here.

### 5.2. Crack Quantification Parameters Updating

As described in Section 2, a detection model $M\left(r^{\prime },\theta \right)$ is needed to show the relation between the damage index and crack length, which can be described by the fitting model shown in Equation (20). The wavelength shift and relative crack length calculated from the measured data of the sensor FBG1 are incorporated to reduce uncertainties through Bayesian updating. Hence, the model is expected to be more reliable for crack size quantification as more relevant data are used to update the baseline model parameters. Based on this type of information, the posterior reads:

(27) $$q\left(\left(\theta ,\sigma_{e}\right)|{\lambda^{\prime }}_{1},{\lambda^{\prime }}_{2},\cdots ,{\lambda^{\prime }}_{n}\right)\propto p(\theta )\times \exp\left(-\frac{1}{2}\sum_{i=1}^{n}{\left[\frac{{\lambda^{\prime }}_{i}-\left(\frac{p_{1}+p_{2}{r^{\prime }}_{i}+p_{3}{r_{i}^{\prime }}^{2}}{1+p_{4}{r^{\prime }}_{i}+p_{5}{r_{i}^{\prime }}^{2}}\right)}{\sigma_{e}}\right]}^{2}\right)$$

The prior distribution of θ is assumed to be a multivariate normal distribution [63]. The mean matrix of this distribution is (1.698,−0.184,0.016,0.013,0.026), and the value of variance is 0.12. To estimate the posterior distribution, each instance of the MCMC simulation has a chain length of 500,000.

**Figure 16:**
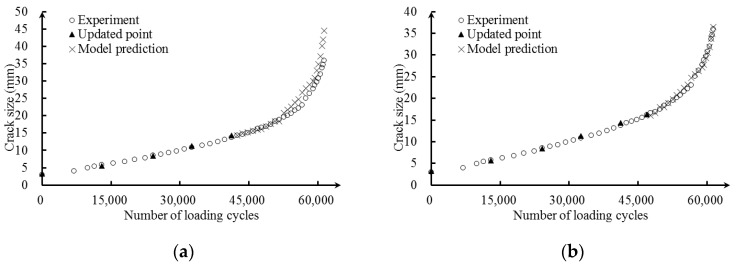
Bayesian updating result with sensor FBG1 measurements. (**a**) Updating with five measurements; (**b**) updating with six measurements.

The updating procedure using measurements is performed incrementally. The measurement here refers to the wavelength shift extracted from the FBG1 reflection intensity spectrum in the fatigue testing and the corresponding crack size. Figure 16 presents the model prediction results of Bayesian updating using the measurements of FBG1. The solid triangles are the data used for updating, and the x-mark denotes the predictions. From Figure 16, it can be observed that the prediction point gradually converges to the actual crack size with additional updating, indicating the effectiveness of the Bayesian updating method in reducing uncertainties. After updating with six measurements, the predicted results match well with the corresponding actual crack size. The resulting detection model has the ability to quantify crack size for the realistic structure. With 500,000 MCMC realizations, a mean vector *u* of these five parameters is obtained and given in Equation (28).

(28) $$u_{\left[p_{1},p_{2},p_{3},p_{4},p_{5}\right]}=\left[1.489,-0.183,0.015,0.019,0.021\right]$$

The updated crack size quantification model is given as:

(29) $$\lambda =\frac{1.489-0.183r^{\prime }+0.015{r^{\prime }}^{2}}{1+0.0195r^{\prime }+0.021{r^{\prime }}^{2}}$$

**Figure 17:**
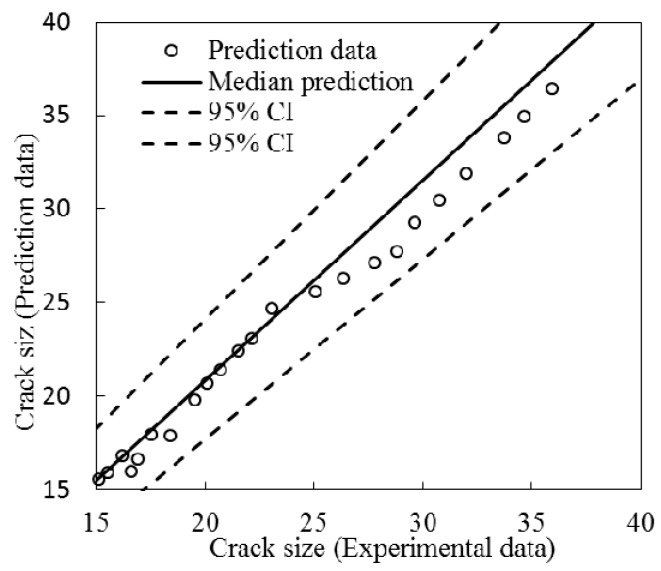
Median and 95% bound predictions using the updated model for FBG1.

To further validate the prediction and updating performance, the median and 95% bound predictions are shown in Figure 17, where the *x*-axis is the measured crack size by optical microscope, and the *y*-axis is the predicted crack size. It can be seen that the median prediction of the model generally characterizes the crack size, and all of the data points are within the 95% prediction bounds.

### 5.3. Crack Detection for FBG2 Sensor

**Figure 18:**
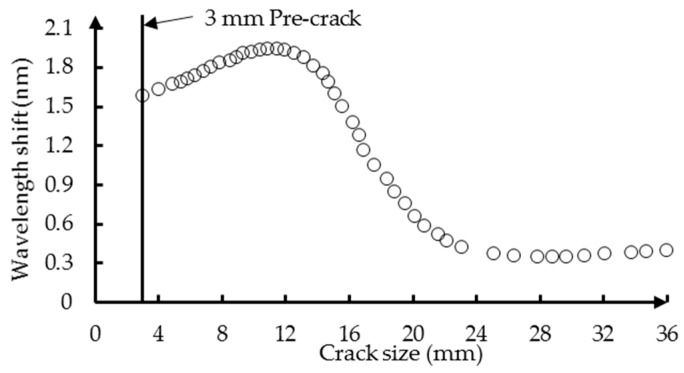
The experiment result: the wavelength shift of FBG2 versus the crack size.

**Figure 19:**
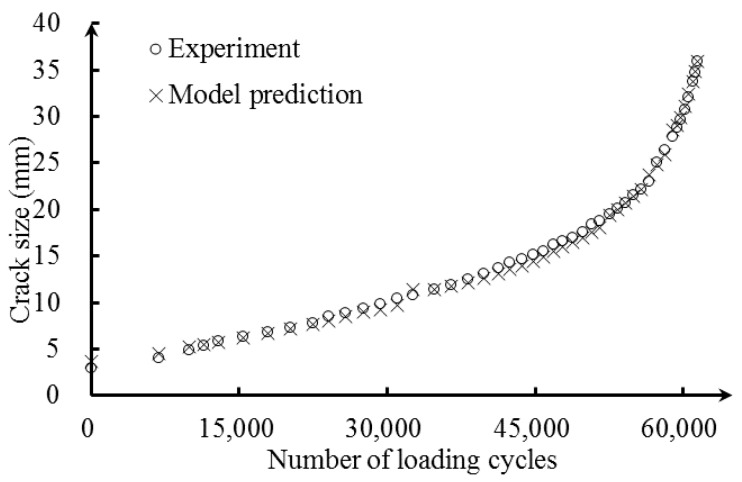
The experimental and model prediction crack size of FBG2 versus the number of loading cycles.

**Figure 20:**
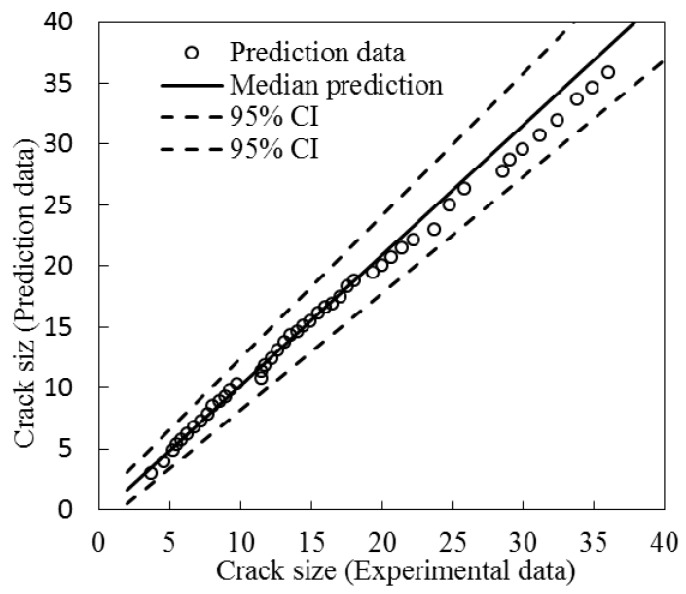
Median and 95% bound predictions using the updated model for FBG2.

The reflection intensity spectrum is affected not only by the size and orientation of the crack defect itself, but also by its distance to the deployed FBG sensors. The FBG2 sensor are placed 15 mm from the hole-edge and 8 mm from the FBG1 sensor, respectively. The crack size versus the wavelength shift of FBG2 is shown in Figure 18. Initially, the wavelength shift increases as the crack propagates and reaches the maximum before the crack reaches the FBG sensor location. Afterwards, the wavelength shift decreases as the crack propagates away from the FBG sensor. The tail of the curve shows smooth changes, which means that the wavelength shift is not sensitive to the crack (in the current situation, the crack size is larger than 24 mm). In this paper, the relative crack size is defined to incorporate the information of FBG sensor location into the detection model. In order to validate applicability of the detection model to an FBG sensor with different locations, the updated model with six measurements of FBG1 in Equation (29) is applied as the detection model for FBG2. The experimental and predicted crack size of FBG2 is presented in Figure 19, where a close agreement is observed. To identify the uncertainty of the model prediction, the median and 95% likelihood function are shown in Figure 20. It can be seen that the model obtained from Bayesian updating with six measurements of FBG1 can reliably quantify the crack size for FBG2.

## 6. Conclusions

This paper presents a general procedure for a probabilistic updating of a crack size quantification method using fiber Bragg Grating sensor and extended finite element method (XFEM). A high-order XFEM method is employed to effectively improve the accuracy of the simulated strain field at the crack region. Based on the simulated strain field, the T-matrix method is used to construct the reflection intensity spectra of FBG sensors, for various crack sizes. The wavelength shift calculated from the reflection intensity spectra is identified as a damage sensitivity index. A baseline model is proposed to describe the correlation between the crack size and the wavelength shift. The Bayesian theory is employed to quantify the uncertainties in realistic applications and to update the detection model. The experimental measurement data from the fatigue testing of an aluminum alloy component is used as the application of the detection strategy proposed. The influence of measurement location is also included. Several conclusions are drawn based on the current study.

- The high-order XFEM shows great potential in structural health monitoring for modeling crack propagation with high accuracy and low computational cost. Combined with the T-matrix method, the reflection intensity spectrum interacting with different crack sizes can be constructed efficiently.
- The damage wavelength shift index is found to be effective for hole-edge crack size quantification. The detection model can yield satisfactory prediction results for FBG sensors with different locations.
- The proposed methodology uses simulation results as a baseline model and incorporates field measurement data to update the model for real structures. The example presented demonstrates the successful applicability of the approach in crack detection.

The procedure described in this study is not limited to a particular model, and other data-driven models and with more complex physics can also be used. It should be noted that all of the FBG sensors are placed on the same side of the pre-crack. Therefore, the updated detection model can be used for FBG sensors with different locations. More complex configurations of FBG sensors are currently in ongoing research. Future studies will also be focused on more robust quantification methods for different crack propagation paths and more complex geometries. The crack generated from both sides of the hole commonly occurs for realistic engineering cases. The prediction model established from FBG1 should be applicable for sensor measurements from the same configuration of crack generated opposite the pre-crack. Uncertainties related with different sensor manufacturing, crack characterization, etc., are reduced through Bayesian updating by incorporating the wavelength shift and relative crack length of sensor FBG1 in fatigue testing. More thorough studies for multiple cracks and connection cracks between two holes are planned as future work.

The FBG sensor senses the strain gradient along the grating due to the stress concentration at the crack tip. The closer the distance to the crack tip, the greater the impact on the reflection intensity spectrum. Therefore, ideally, the sensor should be placed as closer to the hot-spots as possible. However, the crack orientation is usually uncertain for realistic applications. In some extreme cases, the crack will break the FBG sensor. The reliability and durability of sensors is one of the dilemmas for the proposed method and also for the sensor-based structural health monitoring systems. In the current study, sensor location has a rare influence on the performance of the proposed method. Therefore, additional sensors installation can be an option for this problem. The applicability of the proposed method to other structural and material systems, such as composites, also needs further investigations.

## Figures and Tables

**Table 1:** Stress profile $\sigma_{zz}$ and relative error ahead of the crack tip.

Polar Coordinate *r* (mm)	High-Order XFEM		Standard XFEM	
	Normal Stress (MPa)	Relative Error (%)	Normal Stress (MPa)	Relative Error (%)
0.001	11.87	1.29	8.76	27.19
0.01	3.95	0.58	3.19	19.52
0.05	1.80	0.77	1.62	9.10
0.1	1.26	0.24	1.34	5.95
0.3	0.76	2.53	0.83	14.37
0.6	0.50	2.85	0.47	8.94

**Table 2:** Mechanical properties of 7075-T6 aluminum alloy plates.

Material	Elastic Modulus (MPa)	Poisson’s Ratio	Yield Strength (MPa)	Tensile Strength (MPa)
AL7075-T6	73100	0.33	503	572

**Table 3:** Properties of FBG sensors used for fatigue testing.

Parameter	Sensors	
	FBG1	FBG2
Modulation mode of refractive index	Cosine	Cosine
Effective refractive index $n_{eff0}$	1.445	1.465
Poisson’s ratio ν	0.17	0.17
Grating length L (mm)	10.2	10.6
Center wavelength λ (nm)	1529.11	1544.95
Photo-elastic coefficient $p_{11}$	0.113	0.113
Photo-elastic coefficient $p_{12}$	0.252	0.252
